# Supplementary material for: Navigating contradictions in enteric chemotactic stimuli
Source: eLife. 2025 Aug 11;14:RP106261. doi: 10.7554/eLife.106261 (PMC12339005; doi:10.7554/eLife.106261)
Supplement: Supplementary file 1. [file elife-106261-supp1.docx]

**Supplementary File 1.** Summary of prior studies related to indole chemotaxis.

| **Species, Strain(s)** | **Indole Treatment ^a^** | **Reported Response ^b^** | **Experimental Method(s)** | **Chemotaxis & Motility Proteins Involved in Response** | **Ref.** |
| --- | --- | --- | --- | --- | --- |
| *E. coli,* strain RP437 | ≤1 mM  >1 mM | chemorepulsion  chemoattraction | tethered cell assay ^c^ | Tsr, Tar | ^8^ |
| *E. coli,* strain MG1655 | 1 mM ± 0.5-0.1 mM L-Ser | “bet-hedging” ^d^ | diffusion-based assay into long channel with fluorescent imaging | Tar, Tsr | ^9^ |
| *E. coli,* strain RP437 | 500 µM | chemorepulsion, overridden in presence of 500 µM Autoinducer-2 | flow-based microfluidic chemotaxis device coupled to a gradient generator | Tar, Tsr | ^14^ |
| *E. coli,* strain RP437 | ≤1 mM  >1 mM | decreased motility  slower flagellar rotation | tethered cell assay ^c^ | Non-CheY dependent | ^18^ |
| *E. coli*, strain O157:H7 CDC EDL933 | 500 μM | chemorepulsion | agarose plug chemotaxis assay imaged every 5 minutes for 30 minutes | MotB and FliD | ^25^ |
| *S.* Typhimurium, strain ATCC14028s | 1 mM | decreased motility | swimming motility agar plates ^c^ | MotA | ^23^ |
| *S.* Typhimurium, strain ATCC14028s | 1 mM | decreased motility | swimming motility agar plates ^c^ | FlhC | ^24^ |


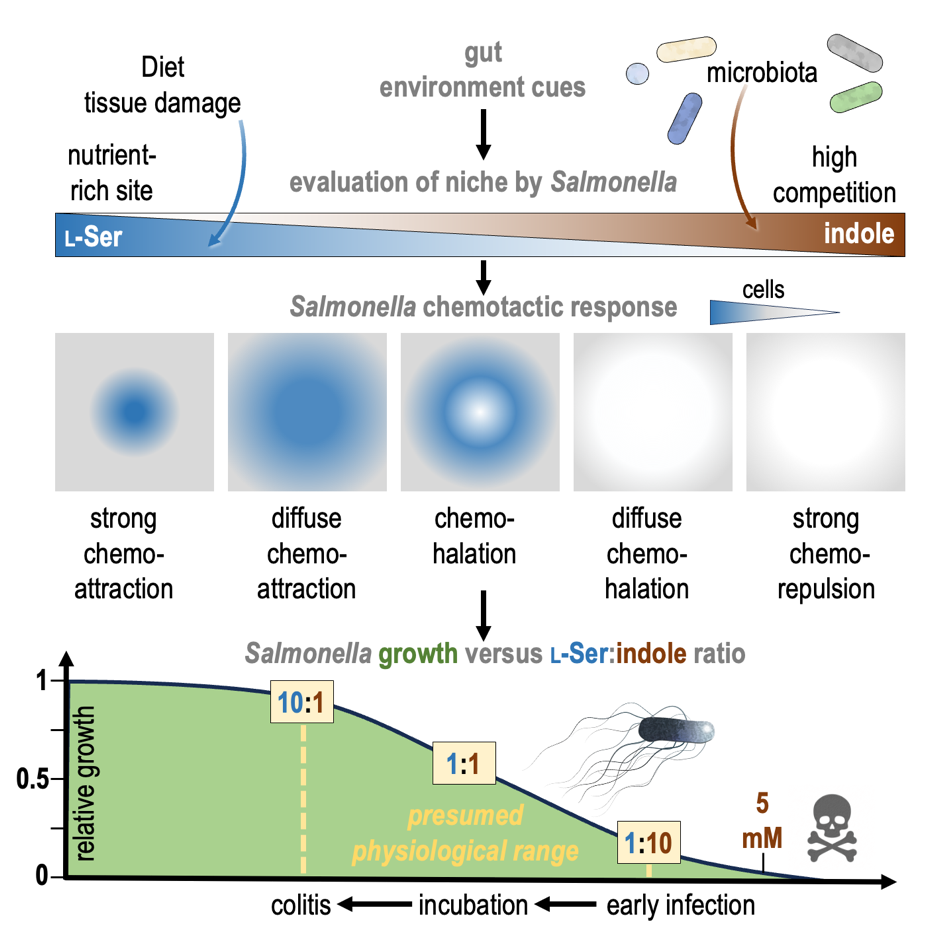


^a^ Source concentration, or ranges, used in experiment.

^b^ The motility or chemotactic response as reported by the study authors; note that some methods employed may not be able to distinguish between responses as a consequence bacterial growth versus chemotaxis.

^c^ The assay may have limitations in its ability to report on either rapid temporal responses, or localization to or from an effector source.

^d^ In this work, we refer to behaviors of this type as “chemohalation” to be similar to the widely-used terms chemoattraction and chemorepulsion.
